# EUPopLink Country report - Turkey

**DOI:** 10.12688/openreseurope.24041.1

**Published:** 2026-07-20

**Authors:** Günce Sabah Eryılmaz, Toygar Sinan Baykan

**Affiliations:** 1 Political Science and International Relations, Bahcesehir University, Istanbul, Turkey; 2 Political Science and Public Administration, Kırklareli University, Kırklareli, Turkey

**Keywords:** populism, euroscepticism, Turkey

## Abstract

This report examines how populism and Euroscepticism intersect in Turkish politics. Turkey’s relationship with Europe has long been shaped by ambivalence, and as EU accession stalled, Euroscepticism spread across the political spectrum. The ruling AKP initially championed EU membership to advance democratic reforms but later shifted toward a civilizational, anti-Western discourse. Other parties — from the Islamist YRP and nationalist ZP on the right to the socialist TİP on the left — also combine populist rhetoric with distinct forms of Euroscepticism. The report traces these positions historically and shows how the “Europe question” has served as a versatile tool for populist actors to redefine political alliances and frame domestic conflicts.

## Plain Language summary

Turkey has long been characterized by a deep ambivalence toward Europe, and Euroscepticism has spread across its political spectrum as EU accession stalled. This country report, prepared as part of the EUPopLink COST Action, traces the relationship between populism and Euroscepticism in the Turkish party system. It introduces the main populist and Eurosceptic actors in Turkey, and examines the nexus between populism and Euroscepticism in the Turkish context. It also considers how the ruling party’s shifting stance on Europe has shaped broader political dynamics and responses to Euroscepticism.

## 1- Introduction

Euroscepticism in Turkey is deeply rooted in the country’s long-standing and often ambivalent relationship with Europe. It is not merely a reaction to the European Union accession process, but rather a broader ideological posture that can be traced back to the late Ottoman modernization period. Europe has consistently appeared as both an admired and distrusted “Other” in intellectual and mass imagination ( Berkes, 2015). The EU integration too, for a very long time, had been part and parcel of discussions around Turkey’s democratization process. However, the EU has considerably lost its normative power in Turkey at least since the Brexit and especially due to the Union’s pragmatic approach to Turkey based on the main strategic target of curbing the immigration. Populist or not, various degrees of euroscepticism grew in the discourses of political parties all over the political spectrum in contemporary Turkey. The present country report was prepared as part of the EUPopLink COST Action ( Andreadis & Vasilopoulou, 2026) following the EUPopLink Theoretical Framework and guidelines ( Lorimer et al., 2026).

## 2- Populism and populist political actors in Turkey

Since 2002, Recep Tayyip Erdoğan’s conservative and populist AKP ( *Adalet ve Kalkınma Partisi* – the Justice and Development Party) has been in power in Turkey. Throughout this process it has become, first, a “dominant party” ( Ayan-Musil, 2015), and then, a “hegemonic” force with clear autocratic proclivities ( Çınar, 2019). During its time in office, the AKP has sidelined various elite veto players (most notably the army), colonized state institutions (most notably judicial institutions), exerted a tight control over media and started to suffocate party competition first, with coopting various smaller parties, and, ultimately, with a full-scale legal assault on the centre-left secular CHP ( *Cumhuriyet Halk Partisi* – the Republican People’s Party). All these developments have made it difficult to call current Turkish political regime even “competitive authoritarian” ( Esen & Gümüşçü, 2016) since it has increasingly started to resemble a more closed autocracy. Populism and discussions around EU also had a crucial role in the transformation of party system and the political regime in Turkey. It is no surprise that rising Euroscepticism has accompanied democratic backsliding in Turkey.

Yet, the origins of populist politics in Turkey go back to much earlier periods of competitive politics, preceding the discussions around EU membership. In the absence of parties strictly representing social divides of Turkey (such as social class differences and ethnic and sectarian identities) populism -with its different ideational, discursive and stylistic reflections- has become a main tool for political elites on the right and left since the transition to a multiparty politics in mid-20 ^th^ century ( Baykan, 2018). Especially after the military coup d’etat in 1980, however, more strictly identity-based parties appeared in the Turkish party system. Islamist and pro-Kurdish parties created significant alternatives on the right and left to catch-all, mainstream, officeseeking parties. The rise of Islamist RP ( *Refah Partisi* - the Welfare Party), the predecessor of Erdoğan’s AKP, was the most notable event during 1990s, which embraced a strictly populist discourse, combined with religious fundamentalism ( White, 2002). The RP was perhaps the most Eurosceptic force Turkish society had ever seen, the founder of which, time and again, had identified the European Union as a “union of Christian states” ( Sayarı, 1996) and frontally rejected Turkey’s attempts to join the EU. It was exactly the disagreement within the growing RP regarding the Turkey’s ideal stance on the EU that paved the way to the rise of initially more moderate AKP from within the Islamist RP ( Öniş, 2001).

The conservative AKP has been the most consistently populist force in Turkish party system since the beginning of 2000s. The party presented itself as the defender of the “humble, conservative, ordinary majority” against the oppression and contempt of “secular and Westernist Kemalist elites” ( Baykan, 2018). In fact, Erdoğan’s AKP has been one of the most important and emblematic cases of populism in the recent decades globally and cross-national studies have demonstrated the rise of populism in Erdoğan’s discourse throughout his terms in office, almost approaching levels of populism that could be seen in Chavez’s speeches ( Hawkins et al., 2019). Also, among the Turkish parties, from a discursive point of view, Erdoğan’s AKP has been the most populist force in Turkey’s national parliament as demonstrated by work deploying quantitative content analysis ( Elçi, 2019).

On the basis of a shared conservative outlook, in the last general and presidential elections in 2023, an Islamist offshoot from the Islamist National View movement ( *Milli Görüş*), the YRP ( *Yeniden Refah Partisi* - the New Welfare Party) led by Fatih Erbakan also supported Erdoğan’s populist AKP. The YRP rapidly became a formidable political organization in recent years based on a widespread organization across Turkey including more than 600.000 members. ^1^ The YRP, which is also identified as a “conspiracy driven party” with a visible populist tone and style, embraced a strictly anti-vaccine, anti-gender equality and anti-LGBT stance and presented all the scientific and progressive measures regarding these issues as conspiracies staged by global Zionist elites ( Oğuz, 2024). In addition, the YRP also constantly deployed a “nativist” language and relied upon a distinction between “native values” and those that are “alien” to Turkish-Islamic culture.

While the main secular left-wing opposition to the AKP, the CHP, can hardly be considered a populist actor in the Turkish party system from a strictly discursive-ideational approach, the left-wing pro-Kurdish DEM Party ( *Halkların Eşitlik ve Demokrasi Partisi* – People’s Equality and Democracy Party- previously HDP - *Halkların Demokratik Partisi*) appears as a major populist actor opposing the AKP. DEM Party, in ideological terms, embraces a mix of ethnic identity politics and socialism. However, alongside embracing Kurdish identity politics, the party also puts emphasis on other marginalized and oppressed groups such as LGBT, women and poor communities across Turkey *vis-à-vis* an oppressive “establishment”. In this respect, from a discourse-theoretical perspective, in various accounts, the HDP has been described as a left-populist entity ( Kaya, 2019; Tekdemir, 2019).

At the opposition front, two remaining forces that should be seriously taken into account with regards to populism are TİP ( *Türkiye İşçi Partisi* – Labour Party of Turkey) and ZP ( *Zafer Partisi* – The Victory Party). The TİP is a left-wing force currently represented in the TBMM ( *Türkiye Büyük Millet Meclisi* - Grand National Assembly of Turkey) (with 3 deputies) that aims the transformation of class-based capitalist order in line with socialism. ^2^ The TİP has been defined as a populist actor in various analyses ( Aksoy, 2024). ^3^ TİP’s program also testify the party’s populist proclivity since it identifies the AKP-led regime in Turkey as the “palace regime” ^4^ that is based on “capitalist classes”. ^5^ In addition, the TİP also states that the character of a future revolution in Turkey should be “populist” and should depend on the working classes supported by “popular sectors”. ^6^

On the radical right, currently, most notable actor in Turkish politics is the ZP led by a former academic Ümit Özdağ (who was, for several months, remained imprisoned due to allegedly “provoking the society to hatred and enmity”). The ZP is a pioneering secular radical right force in Turkey akin to anti-immigration radical right parties of Europe ( Deniz & Kargın, 2023; Koca & Saç, 2024). While currently the ZP is not represented in the parliament, some public opinion research indicates that this new party is receiving considerable support from voters. ^7^ The ZP reveals a deeply ideological, uncompromising Turkish nationalism that puts a particular emphasis on the problem of refugees and immigrants in Turkey. ^8^ The ZP manifesto defines the AKP rule as a “palace regime” and also presents the main opposition CHP as a “yellow opposition” that is in collusion with the ruling party. ^9^ The ZP also locates itself as the representative of ordinary Turkish people *vis-à-vis* the alleged injustice inflicted upon them by “illegal immigrants” protected by the “palace regime”. ^10^ Thus, a deeply conflictual political discourse pitting ordinary Turkish people from all walks of life against an establishment that is allegedly in treason to Turkey characterizes the ZP’s discourse. In this regard, the ZP, indeed, appears as a deeply populist force in contemporary Turkish politics.

This overview of the current effective populist parties also reveals that these parties are populist because they put a strong emphasis on the “people” and simplify the political topography as a vertical conflict between those as the top (elites) and the down (the ordinary majority/the people).

## 3- Positions on ‘europe’ and the populism–Euroscepticism nexus in turkey

Among the parties examined so far only the AKP, YRP, TİP and ZP can be located at the intersection of populism and Euroscepticism since the DEM Party has usually been pro-EU. While the AKP has existed as a political entity since 2001, the others were founded more recently (YRP in 2018, ZP in 2021, and TİP in 2017), suggesting that the populism–Euroscepticism nexus has become more salient in Turkish politics towards the end of the 2010s.

In the 2002 general elections, the AKP openly declared full EU membership as a strategic priority ( Başkan & Gümrükçü, 2012). ^11^ For the AKP, the integration process offered an opportunity to reshape Turkey’s public sphere in a way that would legitimize and normalize the visibility of Islamic identity ( Yılmaz, 2013; Öniş, 2010). In the early 2000s, EU-driven reform packages and the AKP’s populist framing converged in practice. Harmonization laws civilianized the National Security Council and curtailed military–judicial tutelage (e.g., the restructuring/abolition of State Security Courts), which the government presented as EU-mandated democratization while domestically narrating them as removing tutelary “elites” that had thwarted the people’s will ( Çınar, 2008; Öniş, 2010; Saatçioğlu, 2010). By advancing these steps under the banner of Europeanization—expansion of civil liberties, greater cultural pluralism, and the rollback of extra-electoral veto players—the AKP both weakened Kemalist strongholds and deflected accusations of pursuing a religious agenda, recasting liberal reforms as the restoration of popular sovereignty against an exclusionary establishment ( Saatçioğlu, 2014; Kubicek, 2020). However, after 2005, the AKP’s pro-European stance began to fade due to rising Turkey-scepticism within the EU and the bloc’s increasingly ambiguous enlargement agenda. With growing electoral strength in 2007 and 2011, the AKP had consolidated power and no longer relied on the EU reform process to counterbalance the secular establishment ( Aydın-Düzgit & Kaliber, 2016).

The Gezi Park protests in 2013 marked a turning point: the AKP framed EU criticism of its authoritarian response as evidence of European elites conspiring with domestic opponents—particularly Kemalists and liberals—to destabilize the elected government. This gave rise to a full-fledged “Sèvres Syndrome” discourse, casting the EU as a threat to Turkish sovereignty through internal collaborators ( Gülmez, 2020). The narrative draws on a broader historical belief that Western powers have long sought to divide Turkey, as symbolized by the Treaty of Sèvres ( Yılmaz, 2007; Guida, 2008). Following the Gezi Park protests, the AKP abandoned its observer status in the pro-EU European People’s Party (EPP) and joined the Eurosceptic Alliance of European Conservatives and Reformists (AECR), formalizing its shift away from Europe’s political mainstream ( Wódka, 2016).

The 2016 coup attempt further radicalized the AKP’s rhetoric. The EU’s tepid response to the coup attempt was interpreted as implicit support for regime change, while European criticism of post-coup purges reinforced the “West vs. Turkey” dichotomy ( İçener, 2016). By this stage, the AKP had fully embraced a civilizational discourse, positioning Turkey as the leader of the Muslim world against an allegedly hostile Christian club while still nominally supporting accession for strategic leverage, such as migration deals. This ideological shift was operationalized through neo-Ottoman symbolism and Islamist populism, portraying the EU as both culturally alien and politically antagonistic to Turkey’s interests ( Kaya et al., 2020). ^12^

In this context, Euroscepticism became increasingly interwoven with the party’s broader populist strategy, which linked domestic and external “elites” in a unified antagonistic frame. The object of opposition gradually expanded beyond concrete policy disputes to include broader critiques of the principles and future direction of European integration. While the AKP has never formally renounced its goal of EU membership, its rhetoric has increasingly questioned the EU’s normative foundations. The intensity of opposition has fluctuated: early criticisms were largely instrumental and issue-based, whereas later discourse has grown sharper and more emotionally charged, especially around periods of political crisis. Yet this opposition has remained strategic and responsive to domestic priorities, rather than reflecting a fully consolidated ideological rejection of the EU.

Rooted in the *Milli Görüş* tradition, the YRP promotes an Islamist worldview and draws on narratives such as the Sèvres Syndrome, which historically depict Europe as driven by a “Crusader spirit” ( Yılmaz, 2007). Echoing this perspective, the party program expresses strong scepticism toward the EU, accusing some member states of acting on religious bias and reviving a crusader mentality. ^13^ It frames the EU as an external extension of the “alien” cultural elites it opposes domestically, reinforcing its populist–Eurosceptic stance. The object of opposition targets both the normative foundations and political practices of the EU. While the YRP does not explicitly reject EU membership, it sets strict conditions for any potential engagement, insisting on mutual respect and economic cooperation—thereby distancing itself from the broader logic of political integration. ^14^ The party’s opposition can be characterized as hard Euroscepticism, given its categorical rejection of EU values and strong cultural-religious framing.

As a populist radical right party, the ZP portrays the EU as part of an imperialist conspiracy aiming to alter Turkey’s demographic structure and undermine national cohesion, positioning itself as the sole defender of Turkish identity. The party leader Özdağ links the EU, the United States, Israel, and various terrorist organizations as co-sponsors of “strategic migration engineering,” which he describes as a modern form of invasion designed to destroy the Turkish nation from within ( Türk, 2024). The ZP’s historical narrative equates the current refugee crisis with the Crusades, the Sèvres Treaty, and other existential threats from the past, asserting that the nation is once again under siege ( Koca & Saç, 2024). In this context, the party openly calls for the termination of EU accession negotiations. ^15^ While not rejecting cooperation altogether, the ZP proposes replacing integration with a limited, transactional relationship. ^16^ The ZP adopts a hard and consistent Euroscepticism rooted in a conspiratorial, nativist worldview. Framing prolonged existence of refugees in Turkey as a “silent occupation”, it calls for immediate deportation through its “Fortress Anatolia” project ( Koca & Saç, 2024). ^17^

A left-wing populist party, the TİP, views the EU as an instrument of global capitalism and Western imperialism ( Boratav, 2017). The EU is often framed as part of the “Troyka”—a triad of imperialist institutions including the European Commission, IMF, and European Central Bank—particularly in the party’s theoretical publication, *Komünist* ( Özalp, 2017). The TİP’s opposition centers on EU practices such as austerity, labor deregulation, and migration externalization, expressed through a soft but consistent leftist critique of capitalist and imperialist structures. Notably, the 2016 EU–Turkey migration deal is seen as reinforcing both European exploitation and domestic authoritarianism. ^18^ From the TİP’s perspective, the deal reveals the EU as a collaborator of the “palace regime”, deepening the oppression of the Turkish people. The nature of opposition reflects a left-wing populist stance that rejects the EU’s neoliberal model while advocating solidarity with Europe’s working classes. Its Euroscepticism is therefore selective, issue-based, and internationalist rather than nationalist or isolationist.

Since Turkey is not a member, the EU is largely framed as a membership goal rather than a site of political or normative convergence. Questions of institutional depth are postponed. Only parties like the YRP and ZP explicitly reject integration altogether, advocating instead for limited, interest-based cooperation.

## 4- Responses to euroscepticism and consequences

Since the early 2000s, the AKP has played a central role in shaping Turkey’s EU policy—first as a pro-accession force, later as a key source of institutional Euroscepticism. In its early years, the AKP took an adversarial stance toward opposition parties’ sceptical rhetoric, defending EU membership and reforms. However, as the party’s position shifted from a pro-democratic actor to the major autocratizing force, it not only normalized but also amplified Euroscepticism in Turkish politics to legitimize some of its autocratic interventions. More recently, hardline parties like the YRP have pushed the AKP further right on key issues, such as the withdrawal from the Istanbul Convention. Pro-EU actors, including the CHP and the DEM, have largely failed to counterbalance this trend, partly due to the EU’s own ambivalent stance and declining credibility as a political anchor.

## 5- Conclusion

Turkey’s stalled EU accession process offers a unique vantage point on the populism-Euroscepticism nexus. The case’s significance unfolds along three key dimensions. Ideologically, a clear spectrum exists. Right-wing populists (AKP, YRP, ZP) employ a civilisational and nationalist discourse, framing the EU as a culturally alien, Christian club that threatens Turkish sovereignty and Islamic identity, inverting the rhetoric of Europe’s far-right. Conversely, left-wing populists (TİP) critique the EU from an anti-capitalist and anti-imperialist standpoint, viewing it as a neoliberal tool that reinforces exploitation.

Historically, Euroscepticism draws potency from the “Sèvres Syndrome”—the deep-seated fear of Western powers’ attempts to dismantle Turkey. Parties like the AKP and YRP link EU policies to this historical schema, framing criticism as a modern-day existential threat.

Strategically, the case is defined by the ruling AKP’s instrumentalization of its EU stance. It initially championed accession to dismantle the secular Kemalist “elite”, then pivoted to a strategic Euroscepticism, portraying the EU as a hostile force. This demonstrates how the “Europe” issue serves as a versatile tool for redefining political conflict and legitimizing authoritarianism.

In conclusion, Turkey, a Muslim-majority candidate country, enriches the literature by providing a critical contrast to theories derived from member states and underscores how populism-Euroscepticism nexus is shaped profoundly by internal political battles.

## Ethics and consent

Ethical approval and consent were not required.

## Data Availability

No data associated with this article.

## Footnotes

^1^
The exact number is 622.847. https://www.yargitaycb.gov.tr/icerik/1481 accessed:12.5.2025.

^2^

https://tip.org.tr/program/ accessed:14.5.2025.

^3^
See Tanıl Bora’s evaluation of TİP as a left-populist force: https://birikimdergisi.com/haftalik/9586/sol-populizm-i accessed:14.5.2025.

^4^
Refers to the conspicuous presidential palace built in Ankara in 2014 under Erdoğan’s rule.

^5^

https://tip.org.tr/program/ accessed:14.5.2025.

^6^
Ibid.

^7^

https://www.indyturk.com/node/751530/haber/2025%E2%80%99-ilk-kamuoyu-ara%C5%9Ft%C4%B1rmas%C4%B1-yay%C4%B1nland%C4%B1-hangi-parti-ilk-s%C4%B1rada
accessed:14.5.2025.

^8^

https://www.zaferpartisi.org.tr/parti-programi/ accessed:14.5.2025.

^9^

https://www.zaferpartisi.org.tr/kurulus-manifestosu/ accessed:14.5.2025.

^10^
Ibid., 11–12.

^11^

https://www.akparti.org.tr/media/0auje2sn/partiprogrami.pdf accessed:12.5.2025.

^12^
This aligns with the concept of civilizationism, where populist nationalism frames identity conflicts as civilizational struggles. Like Europe’s far-right movements—which weaponize “Christian-secular-liberal” values against Islam ( Brubaker, 2017)—the AKP’s rhetoric inverted this dichotomy. This mirroring underscores how civilizational discourse transcends geographic contexts, serving as a flexible populist tool.

^13^

https://yenidenrefahpartisi.org.tr/page/parti-programi/14 accessed:12.5.2025.

^14^
Ibid.

^15^

https://www.zaferpartisi.org.tr/kurulus-manifestosu/ accessed:14.5.2025.

^16^

https://www.zaferpartisi.org.tr/parti-programi/ accessed:14.5.2025.

^17^

https://www.zaferpartisi.org.tr/anadolukalesiprojesi.pdf accessed:22.5.2025.

^18^

https://tip.org.tr/basin-aciklamalari/6-maddede-turkiye-isci-partisi-gocmen-ve-siginmaci-politikasi/accessed:14.5.2025.

## References

[ref1] Aksoy İU : Siyasal kampanya metinlerinde sol popülizmin dışavurumu: Türkiye İşçi Partisi’nin 2024 yerel seçim bildirgesinde kamulara yönelik olumsallık içeren söylemlerinin analizi. *Yeni Yüzyıl'da İletişim Çalışmaları.* 2024;2(10):38–50.

[ref2] Andreadis I Vasilopoulou S : The populism-euroscepticism nexus in a contested Europe: The EUPopLink COST Action [version 1; peer review: 3 approved]. *Open Res Europe.* 2026;6(27):27. 41710161 10.12688/openreseurope.22680.1 PMC12910195

[ref3] Ayan Musil P : Emergence of a dominant party system after multipartyism: Theoretical implications from the case of the AKP in Turkey. *South European Society and Politics.* 2015;20(1):71–92. 10.1080/13608746.2014.968981

[ref4] Aydın-Düzgit S Kaliber A : Encounters with Europe in an era of domestic and international turmoil: Is Turkey a de-Europeanising candidate country?. *South European Society and Politics.* 2016;21(1):1–14. 10.1080/13608746.2016.1155282

[ref5] Başkan F Gümrükçü SB : Positions of Turkish political parties on European integration. *Southeast European and Black Sea Studies.* 2012;12(1):25–44. 10.1080/14683857.2012.661220

[ref6] Baykan TS : *The Justice and Development Party in Turkey-Populism, Personalism, Organization.* Cambridge University Press;2018. 10.1017/9781108570725

[ref7] Berkes N : *Türk Düşününde Batı Sorunu.* İstanbul: Yapı Kredi Yayınları;2015.

[ref8] Boratav K : Korkut Boratav’la dünyamızın durumu ve solun görevleri üzerine (E. Özalp, Röportaj). *Komünist.* 2017;7:41–54.

[ref9] Brubaker R : Between nationalism and civilizationism: The European populist moment in comparative perspective. *Ethn. Racial Stud.* 2017;40(8):1191–1226. 10.1080/01419870.2017.1294700

[ref10] Çınar M : The Justice and Development Party and the Kemalist establishment. *Secular and Islamic politics in Turkey: The making of the Justice and Development Party.* Routledge;2008; pp.109–131.

[ref11] Çınar K : *The Decline of Democracy in Turkey – A Comparative Study of Hegemonic Party Rule.* London: Routledge;2019. 10.4324/9780429259715

[ref12] Deniz T Kargın İA : Twitter’da Göçmen-Karşıtı Söylemlerin Yükselişi: Zafer Partisi Örneği. *Göç Dergisi.* 2023;10(2):215–231. 10.33182/gd.v10i2.867

[ref13] Elçi E : The Rise of Populism in Turkey: A Content Analysis. *Southeast European and Black Sea Studies.* 2019;19(3):387–408. 10.1080/14683857.2019.1656875

[ref14] Esen B Gümüşçü S : Rising competitive authoritarianism in Turkey. *Third World Q.* 2016;37(9):1581–1606. 10.1080/01436597.2015.1135732

[ref15] Guida M : The Sèvres syndrome and “Komplo” theories in the Islamist and secular press. *Turk. Stud.* 2008;9(1):37–52. 10.1080/14683840701813994

[ref16] Gülmez SB : Rethinking Euroscepticism in Turkey: government, opposition and public opinion. *Ekonomi Politika ve Finans Araştırmaları Dergisi.* 2020;5(1):1–22. 10.30784/epfad.684764

[ref17] Hawkins KA Aguilar R Castanho Silva B : Measuring Populist Discourse: The Global Populism Database. *Paper presented at the 2019 EPSA Annual Conference in Belfast, UK, June 20–22* 2019.

[ref18] İçener E : Turkey–EU relations after the failed July 15 coup attempt. *bilig.* 2016;79:69–87.

[ref19] Kaya A Robert M-V Tecmen A : Populism in Turkey and France: Nativism, multiculturalism and Euroskepticism. *Turk. Stud.* 2020;21(3):361–391. 10.1080/14683849.2019.1637260

[ref20] Kaya M : The potentials and challenges of left populism in Turkey: the case of the peoples’ Democratic Party (HDP). *British Journal of Middle Eastern Studies.* 2019;46(5):797–812. 10.1080/13530194.2019.1634398

[ref21] Koca B Saç S : Korkuyu Politikleştirmek, Öfkeyi Örgütlemek: Fransa’da (Reconquête) ve Türkiye’de (Zafer Partisi) Aşırı Sağ. *Ankara Üniversitesi SBF Dergisi.* 2024;79(2):325–350. 10.33630/ausbf.1203703

[ref22] Kubicek P : Faulty assumptions about democratization in Turkey. *Middle East Critique.* 2020;29(3):245–257. 10.1080/19436149.2020.1770440

[ref23] Lorimer M Roch J Kessel S van : Populism, euroscepticism, and the populism-euroscepticism nexus (Version 1; peer review: awaiting peer review). *Open Research Europe.* 2026;6(73). 10.12688/openreseurope.23109.1 PMC13560596 42728954

[ref24] Oğuz MC : Komplo Güdümlü Siyasal Partilerin Yükselişi ve Türkiye’den Yeniden Refah Partisi Örneği. *Politik Ekonomik Kuram.* 2024;8(3):848–862. 10.30586/pek.1526828

[ref25] Öniş Z : Political Islam at the Crossroads: From Hegemony to Coexistence. *Contemp. Polit.* 2001;7(4):281–298. 10.1080/13569770120106435

[ref26] Öniş Z : Contesting for Turkey’s political “centre”: Domestic politics, identity conflicts and the controversy over EU membership. *J. Contemp. Eur. Stud.* 2010;18(3):361–376. 10.1080/14782804.2010.507919

[ref27] Özalp E : Bunalımlı dönemde iktisat ve siyaset. *Komünist.* 2017;7:27–40.

[ref28] Saatçioğlu B : Unpacking the compliance puzzle: The case of Turkey‘s AKP under EU conditionality. KFG Working Paper Series, 14. 2010.

[ref29] Saatçioğlu B : AKP’s ‘Europeanization’ in civilianization, rule of law and fundamental freedoms: The primacy of domestic politics. *J. Balkan Near East Stud.* 2014;16(1):86–101. 10.1080/19448953.2013.864185

[ref30] Sayarı S : Turkey’s Islamist challenge. *Middle East Quarterly.* 1996;3(3):35–43.

[ref31] Tekdemir O : Left-wing populism within horizontal and vertical politics: the case of Kurdish-led radical democracy in agonistic pluralism. *J. Balkan Near East Stud.* 2019;21(3):335–349. 10.1080/19448953.2018.1497756

[ref32] Türk HB : Populist nationalism and anti-refugee sentiment in Turkey: The case of the Victory Party. *Nationalism and Ethnic Politics.* 2024;30(2):271–295. 10.1080/13537113.2023.2248792

[ref33] White JB : *Islamist Mobilization in Turkey: A Study in Vernacular Politics.* University of Washington Press;2002.

[ref34] Wódka J : Transnational cooperation of Turkish political parties as an ineffective tool of Europeanization. *Southeast European and Black Sea Studies.* 2016;16(2):295–315. 10.1080/14683857.2016.1168640

[ref35] Yilmaz H : Turkish conservatism and the idea of Europe. *Between Europe and the Mediterranean: The challenges and the fears.* Palgrave Macmillan;2007; pp.137–161. 10.1057/9780230287334_10

[ref36] Yilmaz H : Euroscepticism in Turkey: Parties, elites, and public opinion. *Euroscepticism in Southern Europe.* Routledge;2013; pp.185–208.

