# Supplementary material for: EUPopLink Country report - Turkey
Source: Open Res Eur. 2026 Jul 20;6:250. [Version 1] doi: 10.12688/openreseurope.24041.1 (PMC13560853; doi:10.12688/openreseurope.24041.1)
Supplement: Peer review history

# Reviewer report: Reviewer response for version 1

**DOI:** 10.21956/openreseurope.26023.r78498

**Published:** 2026-08-20

**Authors:** Floris Vermeulen

**Affiliations:** 1 Public Administration, University of Twente, Enschede, The Netherlands

Interesting and clear country report on populism in Turkish politics describing the main political parties and the origins of their populist approach. I feel report is almost ready to be indexed.

- I would like to see a short description in the beginning of the report in which populism is defined but more importantly how the authors have identified (in general terms) whether or not a political party or movement is populist or not.
- What are the main criteria and what is the main (secondary) data that is used to identify this.

For the rest I feel the report and the results are ready to be indexed.

Is the case presented with sufficient detail to be useful for teaching or other practitioners?

Yes

Is the work clearly and accurately presented and does it cite the current literature?

Yes

If applicable, is the statistical analysis and its interpretation appropriate?

Not applicable

Are all the source data underlying the results available to ensure full reproducibility?

No source data required

Are the conclusions drawn adequately supported by the results?

Yes

Is the background of the case’s history and progression described in sufficient detail?

Yes

Reviewer Expertise:

Political science with focus on migration and countries of origin.

I confirm that I have read this submission and believe that I have an appropriate level of expertise to confirm that it is of an acceptable scientific standard.

# Reviewer report: Reviewer response for version 1

**DOI:** 10.21956/openreseurope.26023.r78503

**Published:** 2026-08-18

**Authors:** Tugce Ercetin

**Affiliations:** 1 Istanbul Bilgi University, Istanbul, Turkey

The country report successfully scrutinizes the relationship between populism and Euroscepticism in Turkish politics, considering different political party narratives and approaches. The main discussion relies on the understanding and framing of Europe and Euroscepticism in comparative terms, and it also indicates how populist actors and discourse can be articulated.

 I have a few comments, but they are only suggestions, not necessary points. At this point, the authors may evaluate these points:

- The introduction provides an important overview of Euroscepticism in Türkiye. However, it could also engage with the literature on the mutual construction of images and place greater emphasis on this dimension. In other words, a mutual perceived and constructed image can be included. Although Euroscepticism has become particularly visible in recent years in relation to the “deal” and migration, fluctuations in practices concerning “European values” or democratic values could also be addressed.
- I believe that several important points need to be emphasized when categorizing political parties in Türkiye as populist or non-populist. For example, the AKP’s populism has taken different forms of articulation: although it has remained consistent over time, it has also evolved through different issues and narratives, including securitization, the production of negative emotions such as fear, and a more nationalist-Islamist tone. Similarly, the different forms of populism represented by other parties should also be addressed. For instance, can the CHP be categorized as an entirely non-populist party, or is it possible to identify a different form of populism? I believe this discussion could be developed further through closer engagement with the literature. This concerns not only the Özel period; during the local elections, a different form of populism was also performed in opposition to the AKP’s populism. It does not solely suggest making a distinction between left- and right-wing populisms; rather, it can highlight different forms of populism or demonstrate how they differ from each other. Regarding the ZP, greater clarity is needed about the distinction between, and overlap of, nativism and populism. The reasons why the DEM Party is less Eurosceptic than the other parties could be elaborated further, particularly with reference to ECtHR judgments and other democracy-related issues, including human rights and justice. In this sense, a similar position on CHP can be given; of course, it also indicates different approaches within the same political parties. But as I understand, the report focuses on party-level not actors. 
- In the text, various political parties were included in the analysis to give a holistic depiction. From this perspective, the authors may define the selected parties and how they show differences and similarities in terms of analysis of the report. Similarly, the discourse “crusader spirit” belongs to the narrative of YRP, but is it possible to make a distinction with the AKP’s background? Thus, these explanations or short footnotes can be considered; this is not necessary, of course. Additionally, framing as “aliens” has a similar resonation with the AKP’s current “external forces – European voices” articulating cultural perspective, national identity, family, and anti-LGBT issues. To narrate the importance of “native, family and national” values in türkiye, the AKP uses a populist discourse positioning European or Western groups as “enemies”. In this sense, the report may demonstrate how commonalities and differences in Euroscpetic narrative exist. The report clearly and efficiently highlights differences, but some commonalities can also be noted.
- The country name should be shifted to Türkiye.

Is the case presented with sufficient detail to be useful for teaching or other practitioners?

Yes

Is the work clearly and accurately presented and does it cite the current literature?

Yes

If applicable, is the statistical analysis and its interpretation appropriate?

Not applicable

Are all the source data underlying the results available to ensure full reproducibility?

Yes

Are the conclusions drawn adequately supported by the results?

Yes

Is the background of the case’s history and progression described in sufficient detail?

Yes

Reviewer Expertise:

populism

I confirm that I have read this submission and believe that I have an appropriate level of expertise to confirm that it is of an acceptable scientific standard.

# Reviewer report: Reviewer response for version 1

**DOI:** 10.21956/openreseurope.26023.r78502

**Published:** 2026-08-18

**Authors:** Turkay Nefes

**Affiliations:** 1 Spanish National Research Council (CSIC), Madrid, Spain

The report addresses an important case for understanding the relationship between populism and Euroscepticism and brings together useful material on several political actors. However, I believe the current version requires major revision, particularly concerning its structure, conceptual framework, and evidential basis.

 Major comments

 1. The report needs a clearer analytical structure. Political parties currently appear somewhat episodically, without a sufficiently explicit rationale for their selection, order, or relationship to one another. This makes the argument difficult to follow, especially for readers unfamiliar with Turkish politics.

 2. The historical development of Euroscepticism is insufficiently developed. Although the Sèvres Syndrome is mentioned, the report does not systematically trace how dismemberment anxiety, suspicion of European intervention, and related attitudes developed across different Turkish political traditions. As a result, the historical background and contemporary party analysis remain somewhat disconnected.

 One possible way of addressing these first two issues would be to organise the report around broader political traditions—such as Milli Görüş/Islamism, Turkish nationalism, left populism/socialism, and centre-left/centre-right politics—and examine how attitudes towards Europe developed within each. The Sèvres Syndrome could provide one useful historical thread through which both continuities and important differences could be demonstrated. Some of my own work came to mind:

 •    Nefes, T. S. (2024). The impact of group positioning on unfavorable perceptions of Greeks in Turkish politics between 1946 and 1960. Turkish Studies, 25(3), 499–518. https://doi.org/10.1080/14683849.2024.2331244 (Ref 1)

 •    Nefes, T. S. (2015). Understanding Anti-Semitic Rhetoric in Turkey Through The Sèvres Syndrome. Turkish Studies, 16(4), 572–587. https://doi.org/10.1080/14683849.2015.1084876 (Ref 2)

 •    Nefes, T. S. (2013). Political Parties’ Perceptions and Uses of Anti-Semitic Conspiracy Theories in Turkey. The Sociological Review, 61(2), 247–264. (Ref 3)

 I mention these because they came first to my mind. The authors should, of course, feel free to use any other scholarship that they consider more appropriate.

 3. The evidential basis can be more systematic and transparent. The report relies predominantly on secondary studies. This is a legitimate approach for a country report, but its limitations should be acknowledged. It is currently unclear how studies, parties, and examples were selected, and little attention is given to scholarship that might qualify or contradict the interpretations presented. 

 One possible, although entirely optional, solution would be to select representative parties from the main political traditions and examine a comparable source for each—for example, party programmes, manifestos, or another systematically selected body of material. This would provide a clearer comparative basis. 

 4. The key concepts and reporting strategy require clearer definition and justification. Populism, Euroscepticism, and distinctions such as hard and soft Euroscepticism should be defined explicitly. The report should also distinguish more carefully between Euroscepticism, anti-Europeanism, and broader anti-Westernism. This is particularly important when historical attitudes towards “Europe” are used to explain contemporary positions towards the EU.

 The authors should also explain why this particular reporting strategy was selected. At present, the combination of parties, secondary studies, and individual primary sources can appear somewhat convenience-driven. One of the principal theoretical sources used for the EUPopLink framework is itself listed as “peer review: awaiting peer review.” While this does not invalidate the framework, it would be useful to explain its selection and, where possible, anchor the key definitions in established peer-reviewed scholarship.

 5. A table or figure would improve accessibility. A concise table or figure could summarise the principal political traditions or parties, their ideological positions, forms of populism, and attitudes towards Europe and EU integration. This would be particularly valuable for readers unfamiliar with Turkish politics.

 Minor comment

 There is a factual issue concerning YRP. The report states that the party “does not explicitly reject EU membership.” However, some official YRP statements explicitly advocated terminating Turkey's EU accession negotiations. 

 Recommendation

 The Turkish case is highly relevant to the objectives of the EUPopLink project, and the report contains material from which a strong and informative country study could be developed. However, the concerns above affect the organisation of the argument, its historical framework, the selection and comparison of evidence, and the definition and application of its central concepts. I therefore recommend major revisions before approval. The suggestions offered above are intended as possible ways of addressing these issues rather than prescriptions for how the authors should restructure the report.

Is the case presented with sufficient detail to be useful for teaching or other practitioners?

Partly

Is the work clearly and accurately presented and does it cite the current literature?

Partly

If applicable, is the statistical analysis and its interpretation appropriate?

Not applicable

Are all the source data underlying the results available to ensure full reproducibility?

No source data required

Are the conclusions drawn adequately supported by the results?

Partly

Is the background of the case’s history and progression described in sufficient detail?

Partly

Reviewer Expertise:

Turkish politics, intergroup hostility, conspiracy theories

I confirm that I have read this submission and believe that I have an appropriate level of expertise to confirm that it is of an acceptable scientific standard, however I have significant reservations, as outlined above.

## References

[1] : The impact of group positioning on unfavorable perceptions of Greeks in Turkish politics between 1946 and 1960. *Turkish Studies* .2024;25(3) : 10.1080/14683849.2024.2331244499-518 10.1080/14683849.2024.2331244

[2] : Understanding Anti-Semitic Rhetoric in Turkey Through The Sèvres Syndrome. *Turkish Studies* .2015;16(4) : 10.1080/14683849.2015.1084876572-587 10.1080/14683849.2015.1084876

[3] : Political Parties' Perceptions and Uses of Anti-Semitic Conspiracy Theories in Turkey. *The Sociological Review* .2013;61(2) : 10.1111/1467-954X.12016247-264 10.1111/1467-954X.12016

# Reviewer report: Reviewer response for version 1

**DOI:** 10.21956/openreseurope.26023.r77789

**Published:** 2026-08-10

**Authors:** Orçun Selçuk

**Affiliations:** 1 Luther College, Decorah, Iowa, USA

This country report examines the populism-Euroscepticism nexus in Turkish politics. It traces the AKP's trajectory from EU-accession champion in the early 2000s to a civilizational, anti-Western critic. It also situates this alongside three other populist and Eurosceptic actors: the Islamist YRP, the radical-right ZP, and the left-wing TİP. The paper's central contribution is showing how "the Europe question" functions as a flexible discursive tool that different populist actors mobilize toward divergent ends and how this has tracked Turkey's broader democratic backsliding. 

 Here are my comments that would strengthen the quality of the report.

 Substantive Comments

 The authors characterize the current regime as approaching closed autocracy. I agree that the day-to-day developments in Turkey are alarming, but they are not completely outside of the boundaries of competitive authoritarianism. Jailing and harassment of opponents is textbook competitive authoritarianism. Also, arguing that Turkey is a full-scale autocracy undermines the report’s own contribution. The report covers multiple populist parties (YRP, TİP, ZP, DEM) that are not co-opted by the regime and that continue to compete. The persistence of authentic multi-party populist contestation is itself evidence against the closed-autocracy characterization.

 The authors quickly brush off the CHP as a non-populist actor. Although I tend to agree with this characterization for the main opposition party’s general trajectory during the AKP rule, this point is asserted briefly rather than elaborated. One could argue that CHP under Özel (and İmamoğlu) has increasingly used a people-vs-establishment frame. The founding of the Yeni Parti is explicitly framed by Özel as founded "by the people.” This is analytically similar to the populist framing the paper grants to TİP elsewhere in the same section. The authors should specify the criteria that exclude CHP while including TİP as populist. The same question applies to MHP, which is not discussed at all despite being arguably Eurosceptic and having populist features. Given that elements of both populism and Euroscepticism can be identified across nearly the entire party system (we can also add IYI Party here), I'd recommend a comparative table (party × populism indicators × type/intensity of Euroscepticism) to make the coding criteria transparent for the reader. 

 In the conclusion, the report claims that AKP, YRP, and ZP uniformly deploy a "civilisational and nationalist discourse" framing the EU as a "culturally alien, Christian club.” While this generalization applies to AKP and YRP, it does not accurately describe the ZP, which the paper already explains earlier. 

 Points for consideration 

 "Euroscepticism" should also be capitalized consistently. It currently varies across the manuscript.

 Internal party diversity is not addressed. Individual MPs or intra-party factions can hold considerably more (or less) Eurosceptic positions than their party's official platform; treating parties as unitary actors risks overstating coherence. Even a brief acknowledgment would strengthen the analysis.

 The AKP's continued pro-NATO alignment sits in tension with its Eurosceptic and civilizational discourse. Clarifying whether Turkish Euroscepticism (across the parties studied) targets the EU's liberal/normative project specifically, versus Western multilateral institutions more broadly, would sharpen the typology. Some actors in the paper's scope oppose both the EU and NATO, while the AKP is Eurosceptic yet firmly pro-NATO.

 A brief discussion of public opinion or voter-level Euroscepticism for each party (even a sentence per party, where survey data exists) would help ground the elite-discourse analysis in evidence of popular resonance.

Is the case presented with sufficient detail to be useful for teaching or other practitioners?

Yes

Is the work clearly and accurately presented and does it cite the current literature?

Yes

If applicable, is the statistical analysis and its interpretation appropriate?

Not applicable

Are all the source data underlying the results available to ensure full reproducibility?

Yes

Are the conclusions drawn adequately supported by the results?

Yes

Is the background of the case’s history and progression described in sufficient detail?

Yes

Reviewer Expertise:

Populism, Turkish politics

I confirm that I have read this submission and believe that I have an appropriate level of expertise to confirm that it is of an acceptable scientific standard, however I have significant reservations, as outlined above.

# Response

**Published:** 2026-08-13

**Authors:** Günce Sabah Eryılmaz

Dear Professor Selçuk, We would like to thank you for your careful reading of our country report and for your constructive and insightful comments. Your observations raise important points that will strengthen the analysis. We are currently awaiting additional reviewer reports before preparing our revisions, and we will address each of your comments thoroughly in the revised version. With best regards,
